# Hydrodynamic performance assessment of emerged and sub-merged semicircular breakwaters under random waves: An experimental and empirical study

**DOI:** 10.1371/journal.pone.0313955

**Published:** 2025-02-03

**Authors:** Faris Ali Hamood Al-Towayti, Hee Min Teh, Zhe Ma, Idris Ahmed Jae, Agusril Syamsir

**Affiliations:** 1 Department of Civil and Environmental Engineering, Universiti Teknologi PETRONAS, Seri Iskandar, Perak, Malaysia; 2 Institute of Self-Sustainable Building, Universiti Teknologi PETRONAS, Seri Iskandar, Perak, Malaysia; 3 State Key Laboratory of Coastal and Offshore Engineering, Dalian University of Technology, Dalian, Liaoning, China; 4 Institute of Energy Infrastructure, Universiti Tenaga Nasional, Putrajaya Campus, Jalan IKRAM-UNITEN, Kajang, Selangor, Malaysia; Amity University Amity Institute of Biotechnology, INDIA

## Abstract

Mangrove ecosystems and other coastal protection structures are essential barriers protecting coastal populations from the damaging effects of wave energy and increasing sea levels. This study uses a semicircular breakwater (SBW) model in an effort to develop coastal protection measures. The hydrodynamic characteristics of the SBW under random wave conditions, including the transmission coefficient, reflection coefficient, and energy loss coefficient, were thoroughly investigated using physical model experimentation. The main objectives encompass understanding the behavior of the SBW model, developing empirical equations to estimate hydraulic characteristics, and enhancing coastal protection structures to facilitate the preservation and expansion of mangrove ecosystems. Hydrodynamic features of the SBW model were assessed across a spectrum of wave conditions. Experimental testing in a wave flume encompassed a range of relative water depths (*d/h*), including *d/h* = 0.667 for an emerged SBW, *d/h* = 1.000 for an alternatively submerged SBW, and fully submerged conditions for *d/h* = 1.333 and 1.667. Wave steepness (*H*_*i*_*/L*) varied from 0.02 to 0.06, and wave periods ranged from 0.8 to 2.5 seconds. Notably, analysis of an emerged SBW with *d/h* = 0.667 revealed superior wave attenuation compared to *d/h* = 1.000, 1.333, and 1.667 configurations.

## 1. Introduction

**Fig 1:**
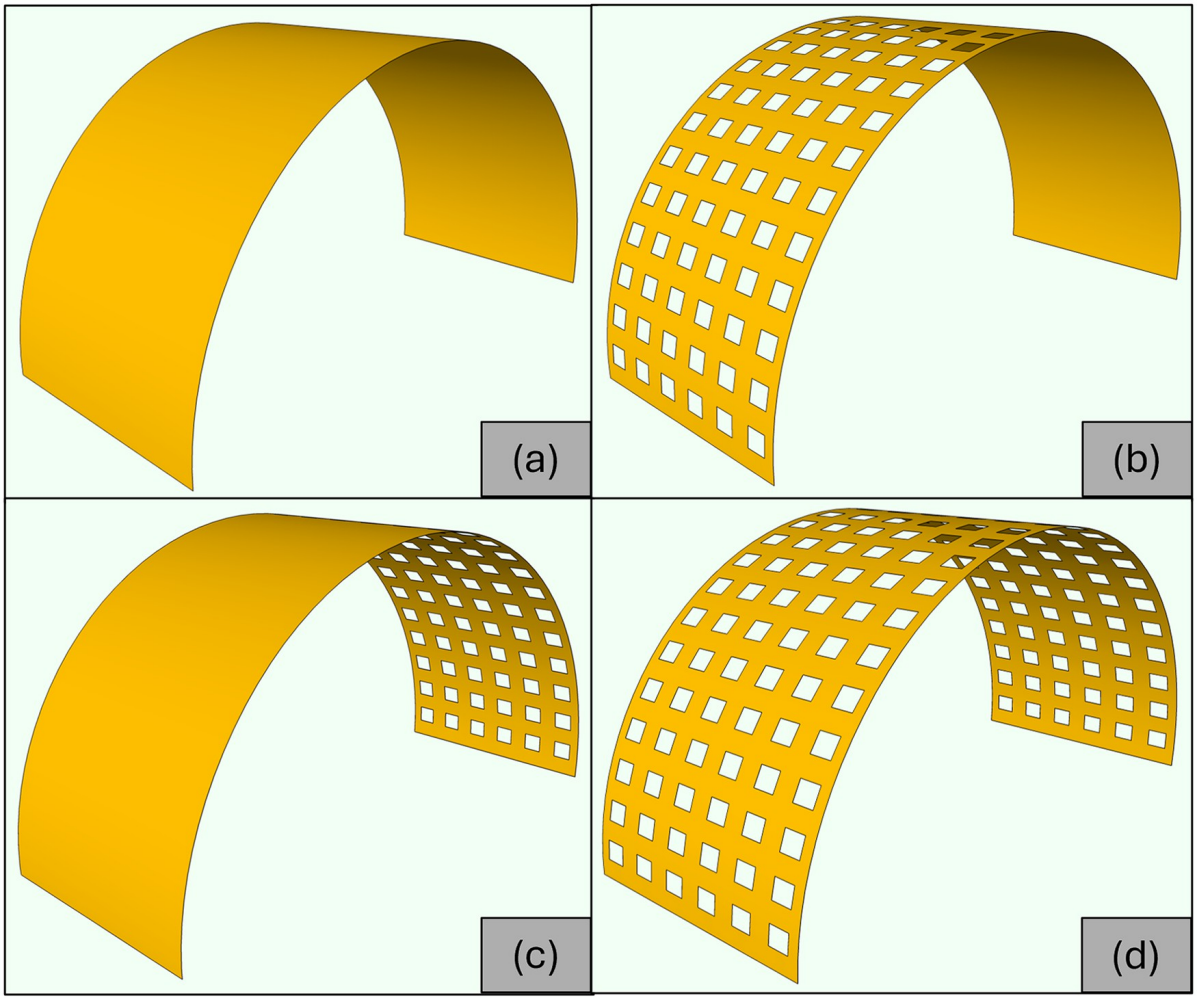
Types of the SBWs—(a) solid SBW, (b) front side SBW, (c) rear side SBW, (d) fully perforated SBW.

The area of the world’s mangrove forests declined by 35% during the latter two decades of the twentieth century [1]. Mangrove forests continue to decline at such alarming rates, particularly in emerging countries, that these ecosystems may completely disappear over the next 100 years [2]. Mangroves in Malaysia are facing alarming rates of destruction and degradation, with a 1% annual destruction rate observed in the Asia-Pacific region [3]. Despite numerous attempts by various agencies and non-governmental organizations (NGOs) to restore mangrove ecosystems through replanting initiatives, the success rate of such efforts has been limited [4]. This limitation stems partly from the need for wave protection at mangrove replanting sites, where mangrove saplings are frequently washed away by the impact of intrusive waves and currents [5–7]. To address this challenge, detached breakwaters offer an alternative approach to mangrove restoration, promoting natural regeneration without the need for extensive planting efforts [4]. Consequently, there is a pressing need for effective coastal engineering structures to provide this protection, with geotextile tubes emerging as one viable option due to their somewhat semicircular cross-section [7]. However, it is important to note that geotextile tubes are susceptible to puncture by sharp objects, and their lifespan typically ranges from 3 to 5 years [8, 9]. The choice of breakwater type depends on various factors, including wave characteristics, water depth, soil foundation conditions, and availability of construction materials and tools. Breakwaters are typically categorized into four groups based on their structural characteristics: sloping (rubble mound), vertical, composite type breakwaters, and special (non-gravity) types of breakwaters such as pile or pneumatic systems [10]. SBWs represent a superior alternative to traditional vertical breakwaters [11]. Their advantages include reduced horizontal wave forces, increased stability against sliding, and lower construction costs. Additionally, the distribution of vertical forces on the foundation soil is nearly uniform, making SBWs advantageous for soft soil foundations. Furthermore, the prefabricated nature of SBWs allows for placement on rubble foundations, eliminating the need for extensive in situ concrete casting work, which is particularly beneficial in rough sea conditions. Moreover, the vault design of SBWs enhances the aesthetic appeal of coastal landscapes [11]. The SBW is classified into four types: solid SBW, front side SBW, rear side SBW, and fully perforated SBW [12]. Fig 1 illustrates these distinct SBW types.

The development and application of SBWs have gained momentum globally, with notable projects in Japan and China showcasing their effectiveness. Japan led the construction of the first SBW at Miyazaki Port from 1992 to 1993, featuring a 36-meter-long structure made of precast reinforced concrete [13]. In China, SBWs have been successfully implemented in various engineering projects, including the 527-meter-long breakwater at Tianjin Port in 1997 and the construction of an 18-kilometer-long semicircular estuary jetty for the Yangtze Estuary Deep Channel Improvement Project in 2000 [14]. A notable advancement in this realm comes from the research conducted by [15], which presents a theoretical solution for the potential flow field of a submerged semi-circular perforated breakwater using the multipole expansion method. This mathematical approach, widely utilized for solving boundary value problems, particularly in cylindrical and spherical wave scenarios, has been extensively detailed in literature such as [16–18]. The analytical model developed by [15] offers calculation techniques for determining the reflection coefficient, transmission coefficient, and wave forces acting on the breakwater. Their derivation process serves as a valuable reference for similar inquiries, including extended investigations into obliquely incident waves [19], flexural-gravity wave scattering by submerged semi-circular ridges [20], Bragg reflection for multiple submerged SBWs [20], and the study of submerged quarter-circular breakwaters [21], among others. Furthermore, a recent study analysed SBWs under regular waves. Using physical models, researchers tested various SBW models with different porosity levels and assessed transmission, reflection, energy dissipation, wave disturbance, and force coefficients. The results of the study indicated that impermeable SBW models were more effective in wave attenuation [22]. Another study employed numerical modelling (FLOW 3D) to assess wave diffraction around submerged and emerged SBW under regular waves. The research found that the wave diffraction coefficient, represented by the *K*_*d*_ value, exhibited a decreasing trend as the relative breakwater width (*B/L* value) increased [23].

This study aims to determine the hydrodynamic characteristics of small-scale SBWs and develop an empirical model for estimating their behavior. By replacing geotextile tubes with robust SBWs, limitations such as short design life and vulnerability to punctures are addressed. SBWs offer a sustainable solution for protecting mangrove ecosystems and have broad applicability in coastal protection. Findings contribute to engineering guidelines for SBWs under random waves, enhancing coastal resilience and biodiversity conservation. The integrated model considers factors like water depth and wave Periods, aligning with prevailing conditions in regions like Malaysia. The paper is structured as follows: Section 1 introduces objectives and background; Section 2 details methodology; Section 3 outlines analytical error analysis; Section 4 presents results and discussion; and Section 5 offers conclusions and findings.

## 2. Methodology

In this study’s procedure, a physical model of the SBW is meticulously fabricated and subjected to testing within a wave tank at the offshore laboratory of Universiti Teknologi PETRONAS. The objective is to capture its hydrodynamic responses under varying wave conditions. Specifically, the transmission coefficient (*C*_*T*_), reflection coefficient (*C*_*R*_), and energy loss coefficient (*C*_*L*_) are computed. Subsequently, the obtained results from the physical tests are rigorously analyzed to assess the SBW’s efficacy in mitigating wave height.

### 2.1 Description of experimental setup

**Fig 2:**
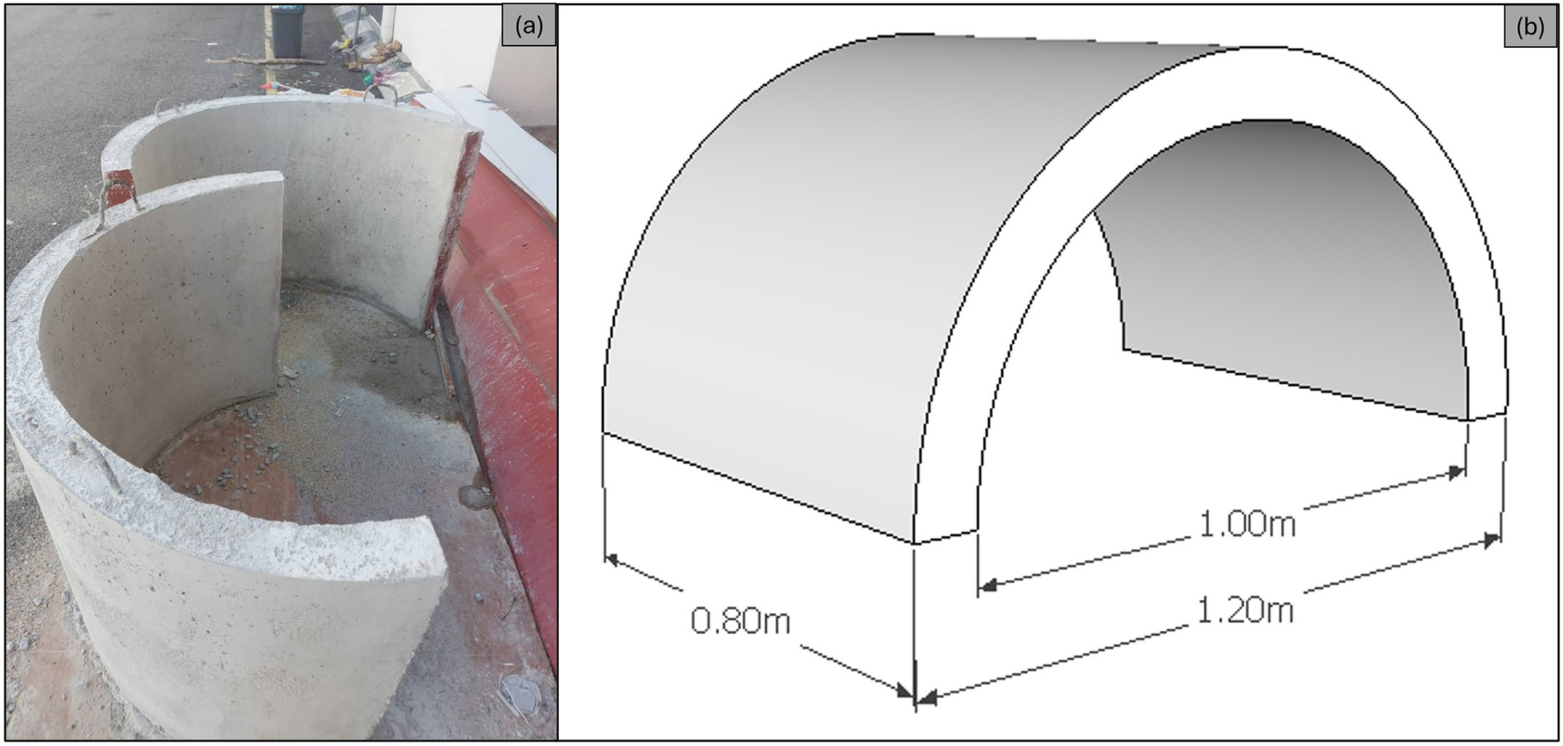
SBW model: (a) Photographic Representation and (b) Schematic Illustration with dimensional annotations.

**Fig 3:**
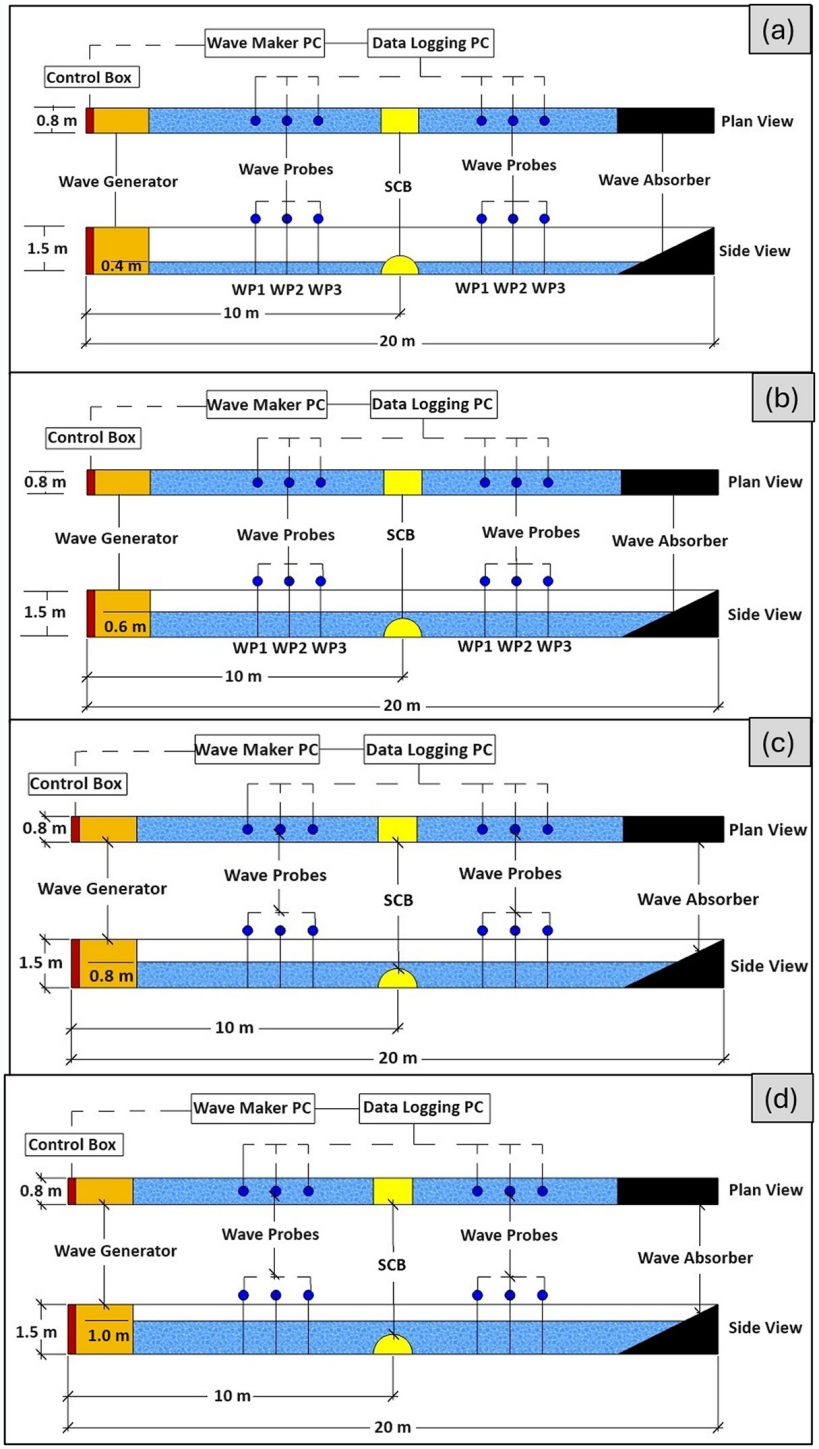
Laboratory setup: (a) *d/h* = 0.667, (b) *d/h* = 1.000, (c) *d/h* = 1.333, and (d) *d/h* = 1.667.

**Fig 4:**
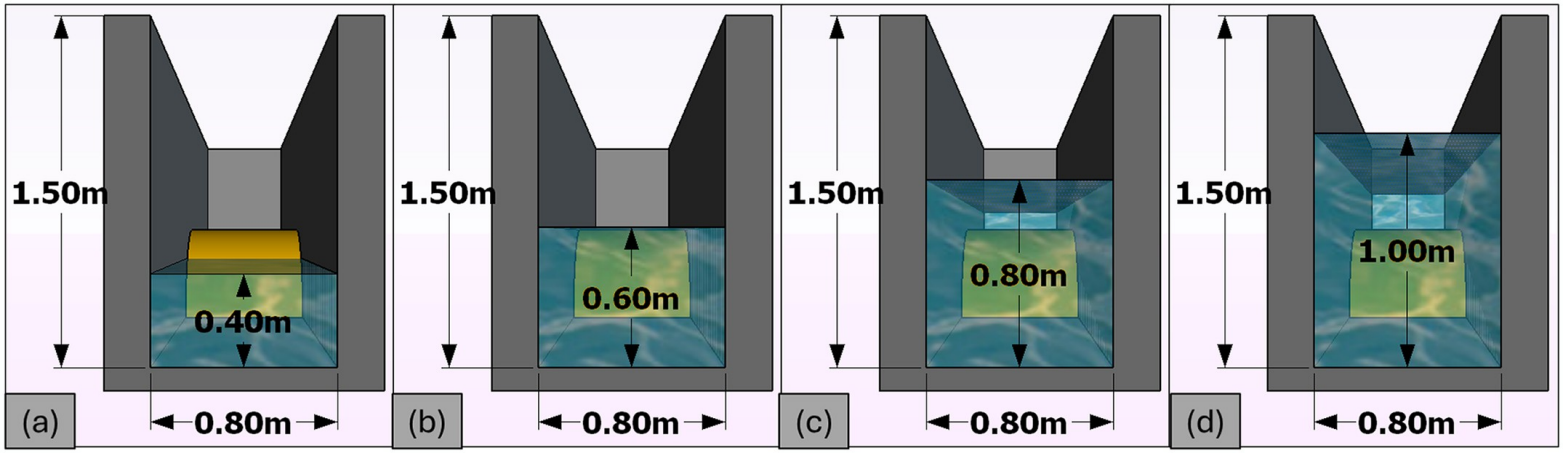
Laboratory cross-section setup with varied *d/h* (a) *d/h* = 0.667, (b) *d/h* = 1.000, (c) *d/h* = 1.333, and (d) *d/h* = 1.667.

**Fig 5:**
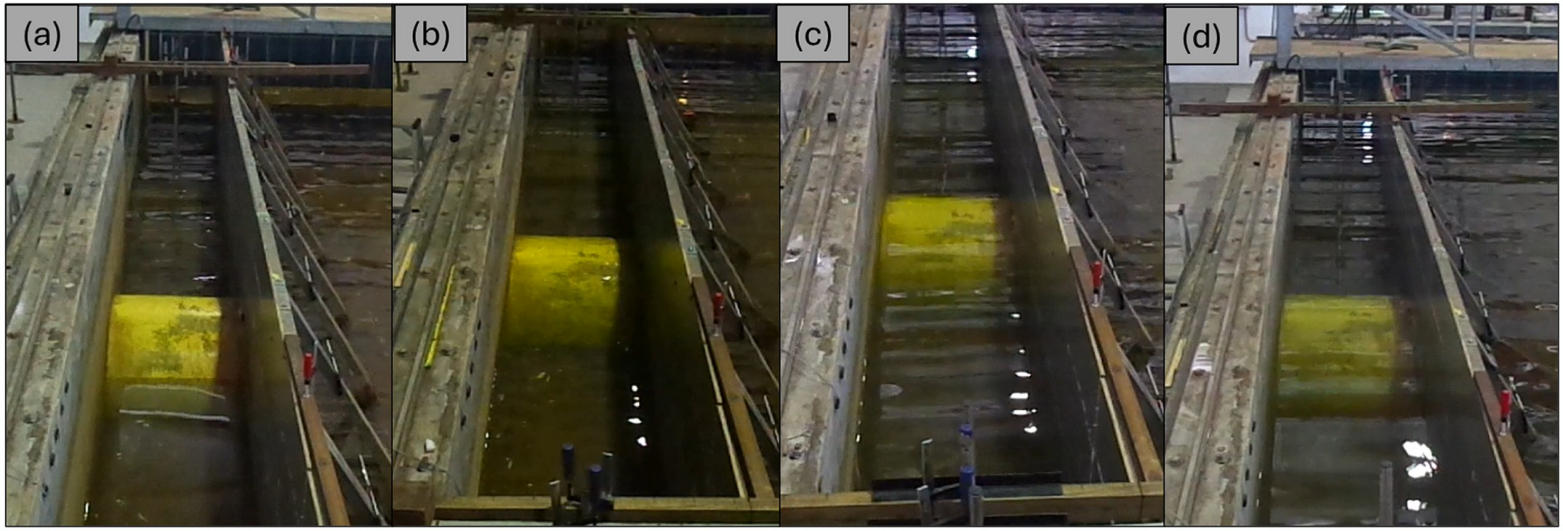
Photograph of laboratory setup at (a) *d/h* = 0.667, (b) *d/h* = 1.000, (c) *d/h* = 1.333, and (d) *d/h* = 1.667.

The study utilized an SBW model that was built with specific dimensions. The model had an external radius (*R*) of 0.6 meters, a thickness of 0.1 meters, and a length of 0.8 meters. The width of the SBW (*B*) was 1.2 meters, while the height (*h*) was the same as *R*. To ensure that the model accurately matched the geometrical requirements of the test facility, a Froude scaling ratio of 1:2.5 was employed. The model was constructed using concrete material with a density of 2400 kg/m3, as shown in Fig 2. A total of 216 experiments were conducted utilizing the least-squares approach of Mansard and Funke. The calibration results for wave probes, are concisely summarized in Table 1. Additionally, Table 2 systematically delineates the scope of test parameters under scrutiny. In Fig 2, the SBW model is represented both photographically (a) and schematically (b), with dimensional annotations for clarity. Fig 3 showcases the laboratory setup, depicting conditions at various *d/h* ratios: (a) *d/h* = 0.667, (b) *d/h* = 1.000, (c) *d/h* = 1.333, and (d) *d/h* = 1.667. Additionally, Fig 4 provides a detailed cross-sectional view of the laboratory setup, highlighting the variations in *d/h* ratios. Further visual context is provided in Fig 5, presenting a photograph of the laboratory setup across different *d/h* ratios: (a) *d/h* = 0.667, (b) *d/h* = 1.000, (c) *d/h* = 1.333, and (d) *d/h* = 1.667. These figures collectively offer a comprehensive portrayal of the experimental setup and conditions investigated in the study.

**Table 1:** Calibration results for wave probes.

Wave Propes	Calibration	Zero	R^2^	95% Calibration @ FSD
WP1	30.5396 mm/V	1.424 V	1.00000	0.0182
WP2	30.4054 mm/V	1.408 V	1.00000	0.0304
WP3	29.9185 mm/V	1.368 V	1.00000	0.0141
WP4	30.2924 mm/V	1.439 V	1.00000	0.0164
WP5	29.4523 mm/V	1.397 V	1.00000	0.0202
WP6	30.4664 mm/V	1.415 V	1.00000	0.0179

**Table 2:** Scope of test parameters.

Wave-specific parameters	Range
Height of incident waves, *H*_*i*_ (m)	0.02–0.38
Water depth, *d* (m)	0.4, 0.6 0.8 and 1.0
Wave period, *T* (s)	0.8–2.5 (*DT* = 0.1s)
Wave steepness *H*_*i*_*/L*	0.02, 0.04, 0.06
Relative water depth, *d/h*	0.667, 1.000, 1.333 and 1.667
Relative wave period, *B/L*	0.2–1.2

### 2.2 Test conditions

The standard JONSWAP spectrum is defined by Eq (1), which determines the spectral density *S*(*f*) at frequency *f* was used to simulate random waves:

$$S\left(f\right)=\frac{\alpha g^{2}}{{\left(2\pi \right)}^{4}}f^{-5}exp\left[-\frac{5}{4}{\left(\frac{f}{f_{p}}\right)}^{-4}\right]\gamma^{exp\left(-\frac{{\left(f-f_{p}\right)}^{2}}{\sigma^{2}{f_{p}}^{2}}\right)}$$ (1)

$$\sigma =\sigma_{a}=0.07\;for\;f\leq f_{p}$$

$$\sigma =\sigma_{b}=0.09\;for\;f>f_{p}$$

This equation governs the spectral density *S*(*f*) at frequency *f*, where various parameters influence its shape and characteristics. These parameters include the Phillips constant *α* = 0.0081, the acceleration due to gravity *g* = 9.81m/s^2^, the frequency of interest *f*, the spectral peak frequency *f*_*p*_, the standard deviation *σ*, and the peak enhancement factor *γ* = 3.3. Depending on the relationship between *f* and *f*_*p*_, the standard deviation *σ* takes on values *σ*_*a*_ = 0.07 for *f* ≤ *f*_*p*_ and *σ*_*b*_ = 0.09 for *f* > *f*_*p*_. These parameters collectively define the spectral density *S*(*f*) and are crucial for accurately simulating random waves. Furthermore, Table 3 presents the temporal variation of the test run time corresponding to changing wave periods.

**Table 3:** Temporal variation of test run time with changing wave period.

Wave period (s)	Test run time (s)
0.8	410
0.9	461
1.0	512
1.1	563
1.2	614
1.3	666
1.4	717
1.5	768
1.6	819
1.7	870
1.8	922
1.9	973
2.0	1024
2.1	1075
2.2	1126
2.3	1178
2.4	1229
2.5	1280

### 2.3 Performance assessment criteria

A part of the energy that waves generate when they interact with breakwaters, regardless of shape, is reflected towards the seaward side of the structures, while another part is dispersed by the structures themselves through energy transformation. The remaining energy is transmitted to the lee side of the structures. This hydrodynamic phenomenon adheres to the principles of energy conservation and can be represented mathematically through the concept of energy equilibrium [24].


$$E_{i}=E_{T}+E_{R}+E_{L}$$ (2)

The symbols *E*_*i*_, *E*_*T*_, *E*_*R*_, and *E*_*L*_ denote the incident, transmitted, reflected, and dissipated energy, respectively. The equation is then elaborated to:

$$1={\left(\frac{H_{T}}{H_{i}}\right)}^{2}+{\left(\frac{H_{R}}{H_{i}}\right)}^{2}+\frac{E_{L}}{E_{i}}$$ (3)

The symbols *H*_*i*_, *H*_*T*_, and *H*_*R*_ denote the wave heights for incident, transmitted, and reflected waves, respectively. Eq (3) can be reformulated as:

$$1={C_{T}}^{2}+{C_{R}}^{2}+{C_{L}}^{2}$$ (4)

The coefficients *C*_*T*_, *C*_*R*_, and *C*_*L*_ represent the transmission, reflection, and energy dissipation, respectively. These coefficients are expressed mathematically as:

$$C_{T}=\frac{H_{T}}{H_{i}}$$ (5)

$$C_{R}=\frac{H_{R}}{H_{i}}$$ (6)

$$C_{L}=\sqrt{1-\;\left(C_{T}^{2}+C_{R}^{2}\right)}$$ (7)

### 2.4 Analytical error analysis

Understanding the interaction between SBW and fluid combinations is crucial in various fields. The coefficient of determination (R^2^) is indeed a crucial metric for assessing the goodness of fit between experimental and empirical results. In a linear regression model, the coefficient of determination (R^2^) is employed to assess the fit [25]. R^2^ quantifies the percentage of the dependent variable’s variance that can be predicted based on the independent variables. A higher R^2^ value indicates a stronger correlation between the data sets, suggesting a better fit of the two outcomes [26]. This is essential in determining the reliability and accuracy of the empirical models in representing the experimental results [27]. In addition to R^2^, various error functions such as root mean square error (RMSE), mean absolute percentage error (MAPE), or other relevant metrics are commonly calculated to quantify the disparities between the experimental and empirical results. Root Mean Square Error (RMSE) gauges the average of the squared differences between experimental and empirical results, offering insight into the accuracy of the model in predicting values. On the other hand, MAPE measures the average of the absolute variations differences between two result values, offering insights into the accuracy of the model in representing the data in percentage terms [27]. Smaller values of RMSE and MAPE indicate a closer agreement between the experimental and empirical values, signifying a more accurate representation of the experimental data by the empirical models [28, 29]. Therefore, in the context of wave steepness and depth of water combinations, the calculation and analysis of these error functions are essential for assessing the accuracy and reliability of the empirical model in capturing the experimental results.

#### 2.4.1 Determination coefficient (R^2^)

The coefficient of determination (R^2^ or r-squared) in regression models is vital for validating empirical results against experimental data. Ranging from 0 to 1, it assesses how well empirical models align with and validate experimental findings. By measuring the proportion of variability in empirical results explained by experimental simulations, R^2^ confirms and validates empirical findings succinctly.


$$R^{2}=1-\;\frac{\sum_{i=1}^{n}{\left(M_{t}-A_{t}\right)}^{2}}{\sum_{i=1}^{n}\left(M_{t}-A_{t}\right)}$$ (8)

#### 2.4.2 Mean Square Error (MSE)

Mean Square Error (MSE) stands as a widely utilized error metric due to its capacity to penalize larger discrepancies more significantly than smaller ones in both empirical and experimental analyses. By squaring errors, larger deviations have a greater influence, shaping the overall error assessment. Computed as the sum of squared differences between empirical and experimental values divided by the number of observations, MSE quantifies the average squared difference between the observed and predicted values, offering a comprehensive measure of the model’s accuracy. The formula for MSE is expressed as follows:

$$MSE=\frac{1}{n}\;\sum_{i=1}^{n}\left(A_{t}-M_{t}\right)$$ (9)

#### 2.4.3 The Root Mean Square Error (RMSE)

The Root Mean Square Error (RMSE), calculated as the square root of the Mean Square Error (MSE), quantifies the typical error magnitude between empirical and experimental values. It serves as a vital metric in validation, indicating how well empirical predictions align with experimental outcomes. The equation for RMSE is as follows:

$$RMSE=\sqrt{\frac{1}{n}\;\sum_{i=1}^{n}\left(A_{t}-M_{t}\right)}$$ (10)

#### 2.4.4 The Mean Absolute Deviation (MAD)

The Mean Absolute Deviation (MAD) serves as a crucial tool in comparing and validating empirical and experimental results. By quantifying the average absolute differences between two sets of values, MAD offers insight into the typical magnitude of discrepancies between the two datasets. This assessment aids in evaluating the accuracy and alignment of empirical predictions with experimental outcomes. The equation for MAD is as follows:

$$MAD=\frac{\sum_{i=1}^{n}\left|A_{t}-M_{t}\right|}{n}$$ (11)

#### 2.4.5 Mean Absolute Percentage Error (MAPE)

The Mean Absolute Percentage Error (MAPE) is vital in assessing accuracy between empirical and experimental results. It computes the average percentage difference between empirical and experimental values, offering a valuable means to gauge alignment. The equation for MAPE is:

$$MAPE=\frac{\sum_{i=1}^{n}\left|\frac{A_{t}-M_{t}}{A_{t}}\right|}{n}$$ (12)

Where: *A*_*t*_ represents experimental data, *M*_*t*_ represents empirical data, *A*_*X*_ signifies the average of experimental data, and n stands for the number of experiments conducted.

## 3. Results and discussion

The experimental investigation undertaken in this study provides valuable insights into wave with SBW interactions. Through meticulous analysis, correlations among essential wave characteristics such as transmission coefficient (*C*_*T*_), reflection coefficient (*C*_*R*_), and energy loss coefficient (*C*_*L*_) were elucidated, considering critical dimensionless parameters like relative wave period (*B/L*), wave steepness (*H*_*i*_*/L*), and relative water depth (*d/h*) across four *d/h* ratios (0.667, 1.000, 1.333, and 1.667), covering emerged, alternatively submerged, and fully submerged configurations. Building upon these experimental findings, empirical model equations were developed and validated against the experimental data. Additionally, the quantitative assessment through metrics such as Mean Squared Error (MSE), Root Mean Squared Error (RMSE), Mean Absolute Deviation (MAD), Mean Absolute Percentage Error (MAPE), and *R*-squared (*R*^*2*^) provided further validation of the empirical models’ robustness across a spectrum of scenarios.

### 3.1 Wave transmission

**Fig 6:**
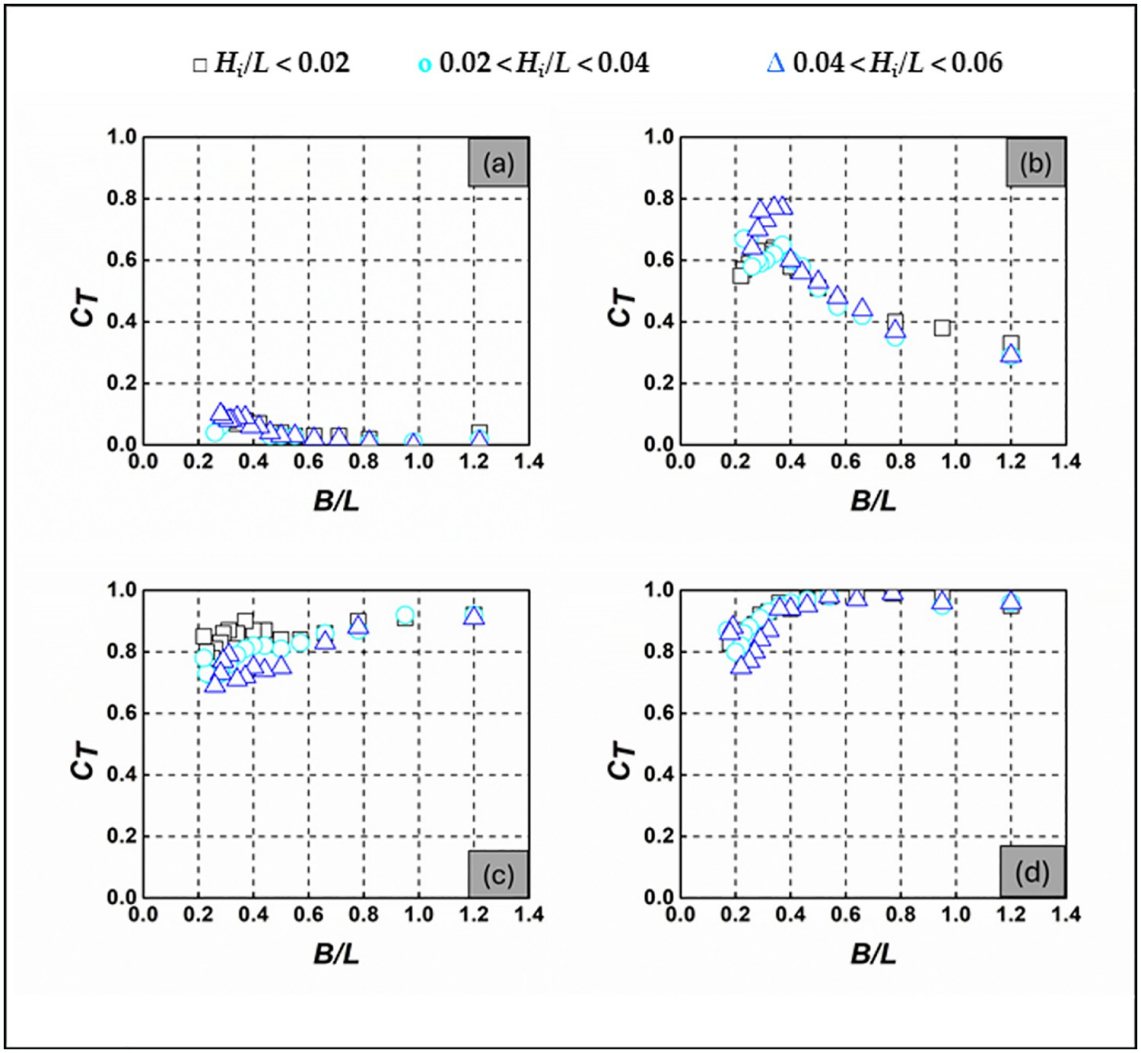
*C*_*T*_ of SBW—(a) *d/h* = 0.667, (b) *d/h* = 1.000, (c) *d/h* = 1.333, and (d) *d/h* = 1.667.

In assessing wave transmission, experimental analyses are conducted to measure the SBW’s ability to convey wave energy. This evaluation primarily relies on the wave transmission coefficient (*C*_*T*_), which is calculated as the ratio of transmitted wave height (*H*_*T*_) to the height of the incident wave (*H*_*i*_) [30–33]. A lower *C*_*T*_ value indicates that more wave energy is being attenuated by the SBW, resulting in increased wave attenuation [30]. Wave transmission involves measuring wave heights to calculate the *C*_*T*_ for the SBW model. The supporting information in S1 Table provides detailed *C*_*T*_ values for various *d/h* ratios and wave conditions shown in Fig 6. Fig 6 illustrates the *C*_*T*_ values for different *d/h* ratios, including 0.667, 1.000, 1.333, and 1.667. Fig 6(a) demonstrates that for the Emerged SBW (*d/h* = 0.667), there is a decrease in the *C*_*T*_ value as the *B/L* ratio increases, regardless of the *H*_*i*_*/L* ratio, *C*_*T*_ values at their maximum and minimum, approximately 0.1 and 0.01 respectively, suggest enhanced wave attenuation as the *B/L* ratio increases. This effect is particularly pronounced for intercepting shorter period waves when *B/L* > 0.4. The main reason for the reduction in wave energy by SBW is mainly due to wave refraction. This phenomenon is especially notable for waves with shorter wavelengths, as they are more sensitive to changes in water depth and interactions with barriers like SBW. Consequently, these waves carry reduced energy and exhibit less pronounced profiles. In Fig 6 (b), the *C*_*T*_ for the alternatively submerged SBW (*d/h* = 1.000) decreases as the *B/L* ratio increases, regardless of the *H*_*i*_*/L* ratio. The recorded maximum and minimum *C*_*T*_ values are approximately 0.77 and 0.29, respectively. This indicates the alternatively submerged SBW’s superior ability to intercept shorter-period waves during random wave actions. Short waves, carrying lower energy flux and exhibiting less development, experience instability near the alternately submerged SBW. Some waves have the capacity to pass over the structure, being transmitted to the sheltered side with reduced wave height. In Fig 6(c), for the fully submerged SBW with a relative water depth (*d/h*) of 1.333, *C*_*T*_ values are relatively high, ranging from 0.69 to 0.92, compared to those observed for the SBW with *d/h* ratios of 0.667 and 1.000. This implies that in comparison to the emerging and alternatively submerged SBW configurations, the submerged SBW exhibits less effective wave attenuation. The submerged SBW is effective in intercepting longer period waves when the *B/L* ratio is less than 0.5. For shorter period waves, the size of the circular water particle orbits decreases as the water column extends in the z-direction [34]. The impact of water particle orbits on the SBW is almost negligible, allowing incident waves to pass through the submerged SBW with minimal flow interference. In Fig 6(d), for the fully submerged SBW with a *d/h* of 1.667, *C*_*T*_ values are relatively high, ranging from 0.75 to 0.99 compared to other *d/h* values (0.667, 1.000, and 1.333), and *C*_*T*_ increases with *B/L*. In Fig 6, wave steepness slightly influences *C*_*T*_ for both *d/h* = 0.667, 1.000, 1.333, and 1.667. *C*_*T*_ values decrease with increasing *H*_*i*_*/L* for *d/h* = 0.667, 1.333 and 1.667. In this scenario, waves of greater steepness are partly intercepted by the SBW, leaving the remaining waves to crest over it, resulting in transmitted waves of significant proportions. As a result, the *C*_*T*_ values for waves with higher steepness are higher.

### 3.2 Wave reflection

**Fig 7:**
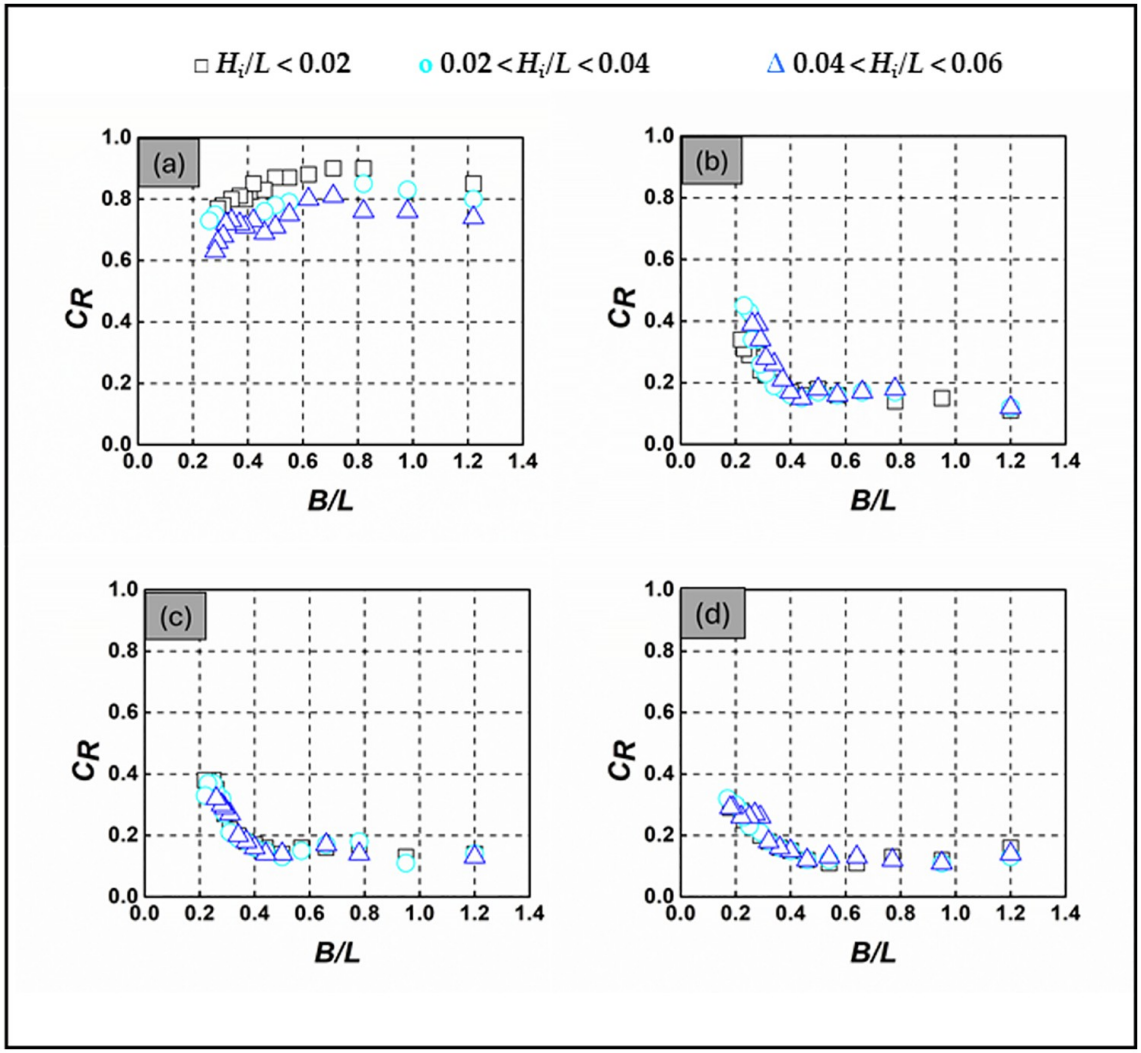
*C*_*R*_ of SBW—(a) *d/h* = 0.667, (b) *d/h* = 1.000, (c) *d/h* = 1.333, (d) *d/h* = 1.667.

Evaluating the SBW’s ability to reflect wave energy is crucial for assessing its effectiveness in coastal protection. This assessment focuses on the wave reflection coefficient, *C*_*R*_, which accurately measures the ratio of the reflected wave height to the incident wave height. This section offers comprehensive insights into the intricate dynamics that govern wave reflection by the SBW model. By meticulously examining various parameters such as wave steepness (*H*_*i*_*/L*), wave period, and water depths, these experiments provide valuable insights into the nuanced behavior of the *C*_*R*_. The assessment of the SBW’s ability to reflect waves is defined as the ratio of the height of the reflected wave (*H*_*R*_) to the height of the incident wave (*H*_*i*_) [33–35]. S2 Table in the supporting information presents detailed *C*_*R*_ values across different *d/h* ratios and wave conditions depicted in Fig 7. Fig 7 meticulously examines the wave reflection characteristics of SBW models, analyzing the influence of *H*_*i*_*/L* and *d/h* ratios. It becomes evident that the variation in *C*_*R*_ concerning *H*_*i*_*/L* is almost imperceptible for the fully submerged and alternatively submerged SBW when *d/h* = 1.000, 1.333, and 1.667, suggesting its insignificance in this context. In contrast, for *d/h* = 0.667, *H*_*i*_*/L* emerges as a significant parameter, with *C*_*R*_ showing an increase as *H*_*i*_*/L* decreases. Looking at specific *d/h* values, the *C*_*R*_ for the emerged SBW (*d/h* = 0.667) demonstrates an increase with increasing *B/L*, displaying relatively high values (ranging from 0.63 to 0.9) compared to *d/h* = 1.000, 1.333, and 1.667. Concurrently, *C*_*T*_ values exhibit an increase with increasing *H*_*i*_*/L* for *d/h* = 0.667 Fig 7(b). The influence of *B/L* on *C*_*R*_ is particularly significant, especially at *B/L* values less than 0.6, where a sharp decline in *C*_*R*_ is observed for alternatively and fully submerged SBW, reaching a minimum at *B/L* > 0.4. The observed effect of *C*_*R*_, as reported in large-scale SBWs [36, 37], highlights the complexity of the relationship. In summary, the proposed SBW exhibits high reflectivity (0.1 < *C*_*R*_ < 0.45) against longer waves (*B/L* < 0.4) for *d/h* = 1.000, 1.333, and 1.667. Comparing the *C*_*R*_ graphs in Fig 7 indicates that wave reflection by the emerged SBW is more prominent than the alternatively submerged SBW. This is likely due to its greater disruption of incident waves. The notable wave reflection characteristics of the SBW within the lower *B/L* range in Fig 7(b)–7(d) lead to reduced wave transmission. In conclusion, the proposed SBW proves to be a strong reflector for *d/h* = 0.667 across all *B/L* values and only for *B/L* less than 0.4 for *d/h* = 1.000–1.667. With an improvement of approximately 15% to 25%, the emerged SBWs outperform the submerged SBW in terms of reflective performance.

### 3.3 Wave energy loss

**Fig 8:**
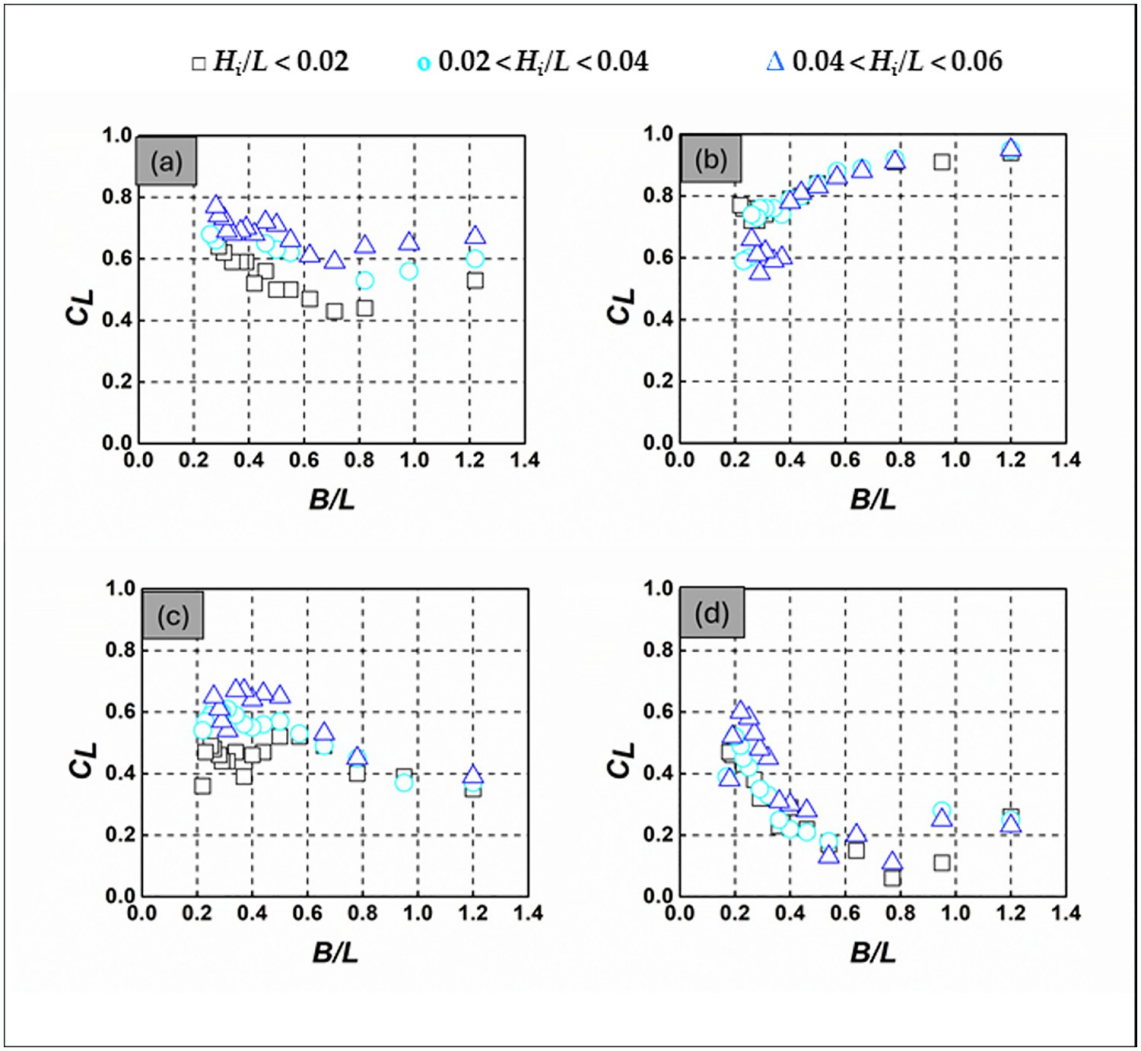
*C*_*L*_ of SBW—(a) *d/h* = 0.667, (b) *d/h* = 1.000, (c) *d/h* = 1.333, (d) *d/h* = 1.667.

Analyzing the dissipation of wave energy linked with SBW models is a crucial element in comprehending their effectiveness as coastal protection methods. The efficacy of the SBW is assessed through its wave reflection, transmission, and energy dissipation characteristics [38]. The wave dissipation coefficient, *C*_*L*_, acts as a gauge of hydraulic efficiency, derived from the energy conservation law [32, 34, 37, 39–41]. Assessing the effectiveness of SBW in mitigating wave energy is crucial for evaluating their suitability as coastal defense solutions. SBWs, characterized by their curvature, demonstrate significantly higher energy dissipation compared to conventional types [42]. Fig 8 depicts the *C*_*L*_ variation across SBWs with different *B/L* and *H*_*i*_*/L* under random wave conditions. Emerged and fully submerged SBWs generally show less wave energy attenuation compared to alternatively submerged SBWs, mainly due to their pronounced curvature, especially noticeable for *d/h* = 1.000. Interestingly, the alternatively submerged SBW with *d/h* = 1.000 effectively intercepts incoming waves, leading to enhanced energy dissipation as waves traverse the crest. This effect is highlighted in Fig 8(b), which demonstrates a significant increase in *C*_*L*_ with *B/L*, particularly for shorter wavelengths, indicating efficient energy release upon wave impact. Conversely, for *d/h* = 0.667, 1.333, and 1.667, *C*_*L*_ decreases with increasing *B/L*, showing varied behavior depending on relative water depth. Moreover, *C*_*L*_ values for *d/h* = 0.667 (Fig 8(a)) range from 0.43 to 0.77, for *d/h* = 1.000 (Fig 8(b)) from 0.55 to 0.95, *d/h* = 1.333 (Fig 8(c)) between 0.35 and 0.67, and for *d/h* = 1.667 (Fig 8(d)), from 0.06 to 0.6. The influence of *H*_*i*_*/L* on *C*_*L*_ varies across different *d/h* and *B/L* values, with distinct trends observed for *d/h* = 0.667, 1.333, and 1.667, primarily driven by wave breaking over the SBW. In summary, the alternatively submerged SBW (*d/h* = 1.000) shows superior wave dissipation performance compared to fully submerged SBWs (*d/h* = 1.667 and 1.333), with emerged SBWs at *d/h* = 0.667 demonstrating similar efficacy. S3 Table in the supporting information provides a comprehensive set of *C*_*L*_ values for different *d/h* ratios and wave conditions shown in Fig 8.

### 3.4 Empirical analysis

The hydraulic performance of the SBW model depends on its geometry, wave characteristics, and water depth. The relative SBW width (*B/L*), the relative water depth (*d/h*), and the wave steepness (*H*_*i*_*/L*) are three non-dimensional characteristics associated with the hydraulic coefficients that are identified and stated as follows:

$$\left.\begin{array}{c} C_{T}\\ C_{R}\\ C_{L} \end{array}\right\}=f\left[\frac{B}{L},\;\frac{H_{i}}{L},\;\frac{d}{h}\right]$$ (13)

Several empirical formulas were constructed utilizing several regression methods to forecast the SBW model’s overall hydrodynamic performance. The current study examines the behavior of SBW under random wave conditions, whereas earlier research concentrated on regular waves [22], Nonetheless, the empirical correlations obtained from earlier research [22] remain pertinent and serve as a foundation for determining the *C*_*T*_, *C*_*R*_, and *C*_*L*_ coefficients in Eq (12). A consistent and knowledgeable method of forecasting hydrodynamic performance is ensured by this research, which acknowledges earlier discoveries and applies them to the particular context of random waves.


$$\left.\begin{array}{c} C_{T}\\ C_{R}\\ C_{L} \end{array}\right\}=f\left[\frac{B}{L},\;\frac{H_{i}}{L},\;\frac{d}{h}\right]=f\left[\prod_{1},\;\prod_{2},\;\prod_{3}\right]$$ (14)

The following are the general prediction formulas for *C*_*T*_, *C*_*R*_, and *C*_*L*_:

$$\begin{array}{c} \left.\begin{array}{c} C_{T}\\ C_{R}\\ C_{L} \end{array}\right\}=x_{1}\prod_{1}+x_{2}\prod_{2}+x_{3}\prod_{3}+x_{4}{\prod_{1}}^{2}+x_{5}{\prod_{2}}^{2}+\\ x_{6}{\prod_{3}}^{2}+x_{7}\prod_{1}\prod_{2}+x_{8}\prod_{1}\prod_{3}+x_{9}\prod_{2}\prod_{3}+x_{10} \end{array}$$ (15)

Table 4 lists the coefficients for each of the terms in Eq (15).

**Table 4:** Empirical coefficients for *C*_*T*_, *C*_*R*_ and *C*_*L*_.

	*C*_*T*_	*C*_*R*_	*C*_*L*_
*x*_*1*_	-0.450	-0.346	-0.021
*x*_*2*_	-0.590	1.793	-0.208
*x*_*3*_	2.879	-3.252	1.536
*x*_*4*_	-0.063	0.351	0.235
*x*_*5*_	26.411	-50.954	21.720
*x*_*6*_	-0.913	1.176	-0.777
*x*_*7*_	2.182	-0.477	-2.227
*x*_*8*_	0.373	-0.199	-0.263
*x*_*9*_	-2.208	1.416	1.386
*x*_*10*_	-1.336	2.512	-0.031

It is crucial to emphasize that the following conditions must be met in order for the suggested empirical equations to be applicable:

$$0.5<\frac{d}{B}<1.25$$

$$0.10<\frac{B}{L}<1.2$$

$$0.01<\frac{H_{i}}{L}<0.06$$

$$0.667<\frac{d}{h}<1.667$$

**Fig 9:**
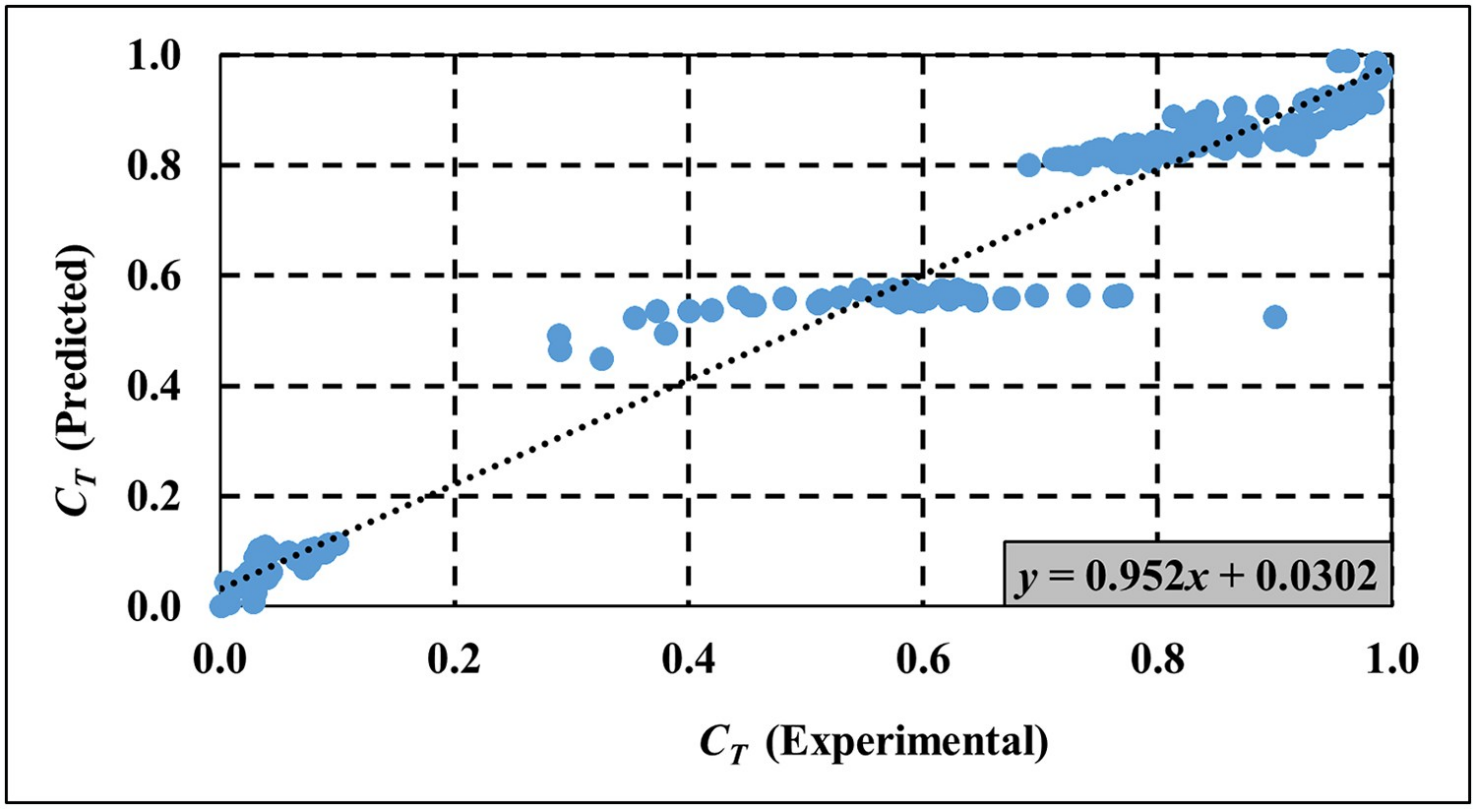
Validation of empirical model for *C*_*T*_ prediction.

**Fig 10:**
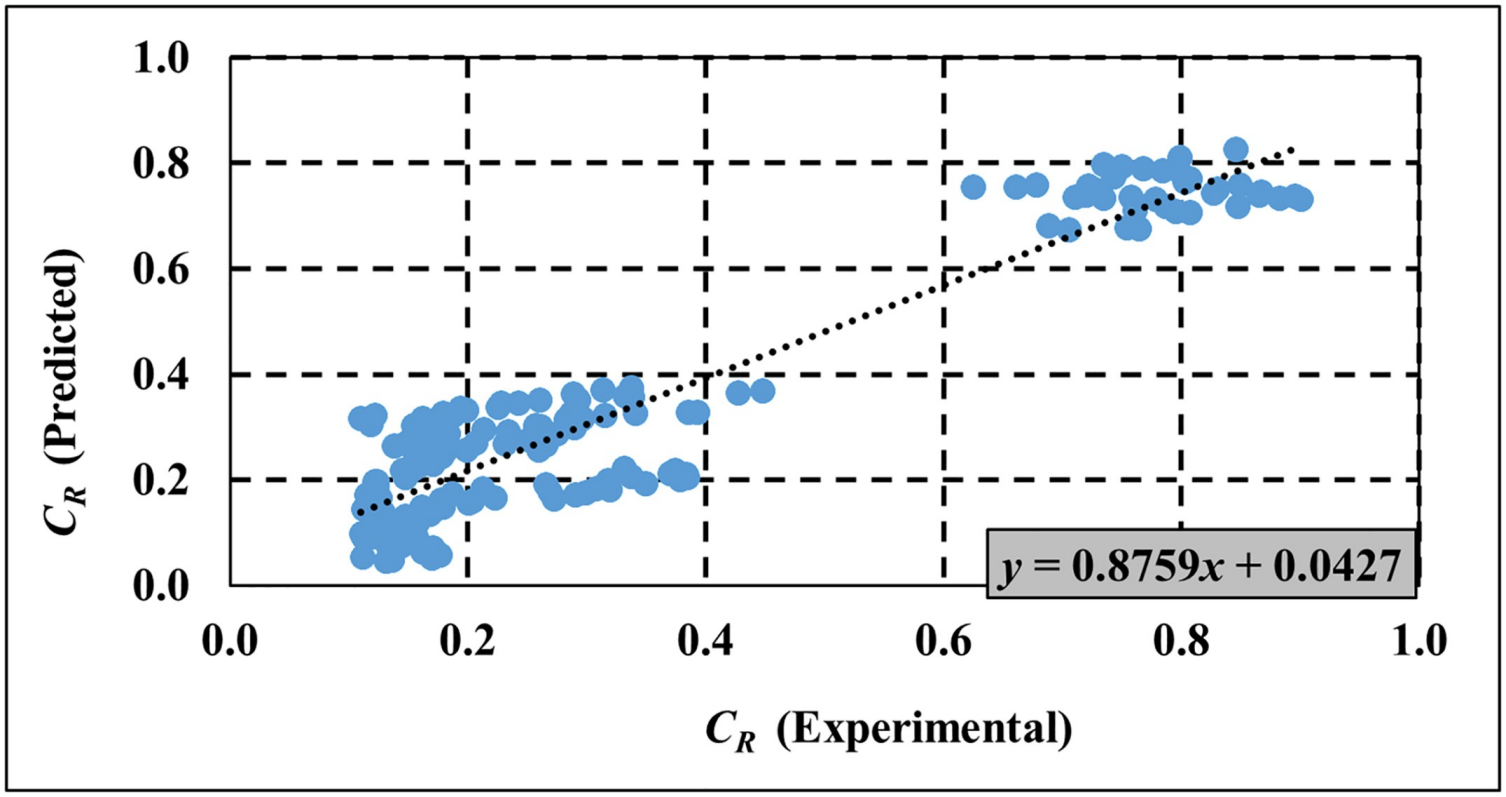
Validation of empirical model for *C*_*R*_ prediction.

**Fig 11:**
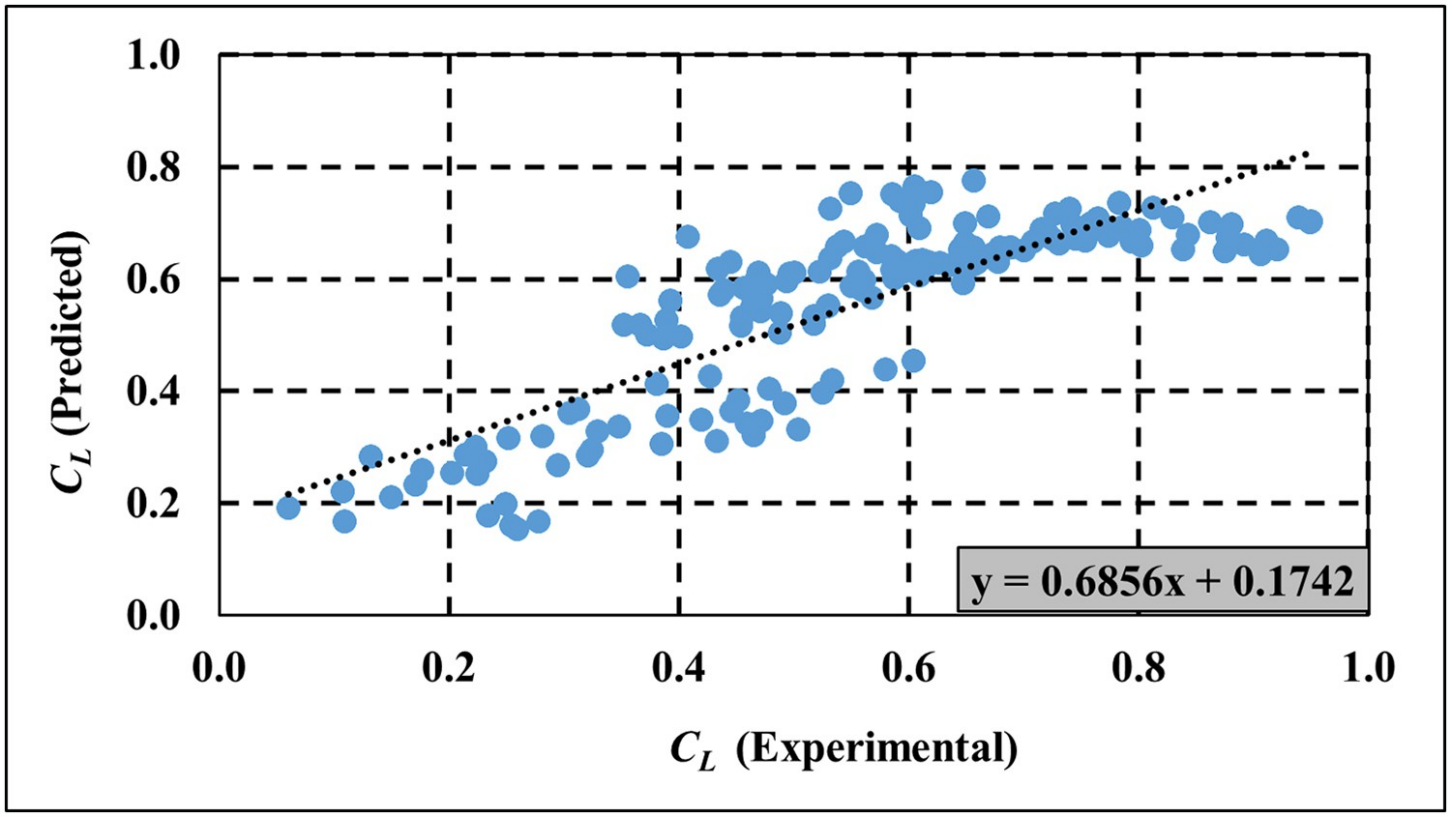
Validation of empirical model for *C*_*L*_ prediction.

The experimental results in Figs 9–11 are used to validate the computed results from the empirical models for *C*_*T*_, *C*_*R*_ and *C*_*L*_ respectively. Table 5 provides a comprehensive analysis of key performance metrics for the SBW, focusing specifically on *C*_*T*_, *C*_*R*_, and *C*_*L*_. The remarkable alignment observed between empirical model predictions and experimental data underscores the accuracy of the empirical model in predicting wave characteristics. Supporting S4–S6 Tables provide detailed empirical data for *C*_*T*_, *C*_*R*_, and *C*_*L*_, respectively, used in Figs 9–11. Meanwhile, S7–S9 Tables present both experimental and empirical data for the calculated error analysis of *C*_*T*_, *C*_*R*_, and *C*_*L*_, as shown in Table 5. Key metrics such as Mean Squared Error (MSE), Root Mean Squared Error (RMSE), Mean Absolute Deviation (MAD), Mean Absolute Percentage Error (MAPE), and R-squared (R^2^) values further validate the model’s precision in capturing *C*_*T*_, *C*_*R*_, and *C*_*L*_. Lower values for MSE, RMSE, MAD, and MAPE are desirable as they indicate minimal prediction errors, while higher R^2^ values signify a better. Overall, the proposed empirical formulas for the respective SBW model demonstrate good estimation of *C*_*T*_, *C*_*R*_, and *C*_*L*_.

**Table 5:** Evaluation parameters for empirical models.

	MSE	RMSE	MAD	MAPE	R^2^
*C*_*T*_	0.01	0.07	0.05	0.37	0.98
*C*_*R*_	0.01	0.09	0.07	0.32	0.96
*C*_*L*_	0.01	0.11	0.09	0.19	0.92

Table 6 presents the significance of independent variables in the analysis. Each variable, labeled as ∏_1_, ∏_2_, and ∏_3_, is assessed for its contribution to the predictive power of the model. Notably, ∏_3_ emerges as the most influential variable, with an importance value of 0.770. In contrast, ∏_1_ and ∏_2_ display lower importance values of 0.146 and 0.084, respectively. This provides insight into the relative significance of each variable in predicting the outcome.

**Table 6:** Independent variable importance.

∏_1_	0.146
∏_2_	0.084
∏_3_	0.770

**Fig 12:**
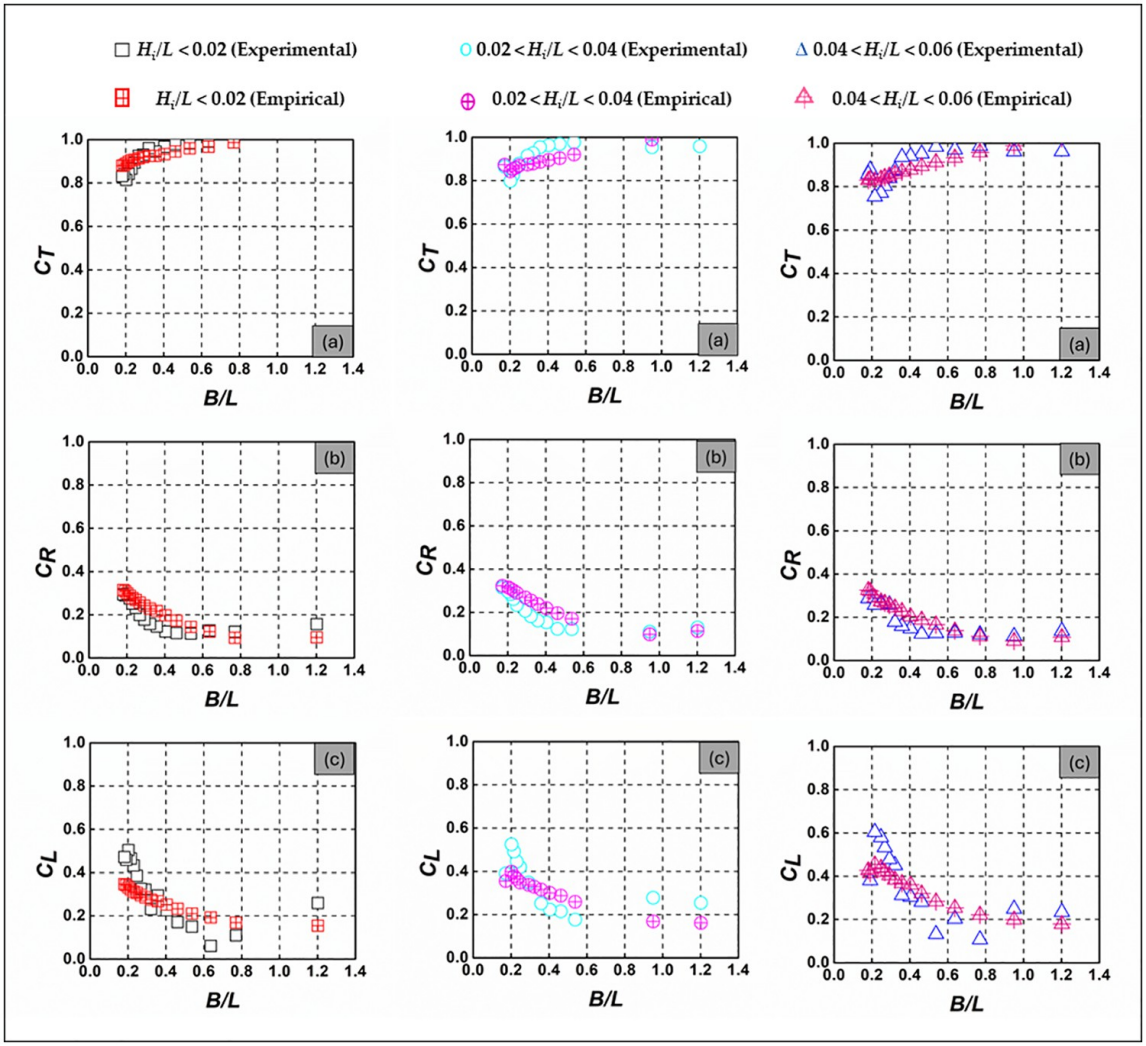
Comparison of experimental and empirical a) *C*_*T*_, b) *C*_*R*_, and c) *C*_*L*_ for *d/h* = 1.667.

Fig 12 presents a comprehensive validation of the *C*_*T*_, *C*_*R*_, and *C*_*L*_ for submerged SBW across varying wave steepness conditions *H*_*i*_*/L* < 0.02, 0.02 < *H*_*i*_*/L* < 0.04, and 0.04 < *H*_*i*_*/L* < 0.06) at a relative water depth of *d/h* = 1.667. This depiction provides valuable insights into the empirical model’s predictive accuracy, effectively capturing the nuanced behavior of *C*_*T*_, *C*_*R*_, and *C*_*L*_. Notably, the empirically derived *C*_*T*_, *C*_*R*_, and *C*_*L*_ values from SPSS-generated equations demonstrate a remarkable alignment with experimental findings, highlighting the reliability and accuracy of the empirical approach in predicting these coefficients. Supporting S7–S9 Tables present comprehensive experimental and empirical data for *C*_*T*_, *C*_*R*_, and *C*_*L*_, respectively.

## 4. Conclusions

In conclusion, this study introduces and comprehensively evaluates small SBW designs customized for diverse coastal protection applications. The hydraulic characteristics of these SBWs were extensively investigated using physical modeling. Notably, the analysis of the *C*_*T*_ revealed nuanced effectiveness among different SBW configurations, with the emerged SBW (*d/h* = 0.667) showing a smaller *C*_*T*_ compared to *d/h* = 1.000, *d/h* = 1.333, and 1.667. By correlating *C*_*T*_ values with *B/L* ratios and meticulously comparing transmitted wave height with permissible criteria for mangrove survival, the efficacy of SBWs at different *B/L* dimensions was assessed. The SBW achieved a reflection accuracy of nearly 90% when *d/h* equaled 0.667. The alternatively submerged SBW (*d/h* = 1.000) demonstrated superior wave attenuation performance, outperforming the fully submerged SBW (*d/h* = 1.667) with a substantial 70% reduction in incident wave height when exposed to random waves with shorter periods. However, it’s important to note that wave energy dissipation tended to worsen as the immersion depth increased. Empirical results closely aligned with experimental findings, demonstrating the effectiveness of empirical model equations in the estimation of the hydraulic characteristics of SBW.

## Supporting information

S1 Table Experimental transmission coefficient (*C*_*T*_) for different *d/h* ratios. (DOCX)

S2 Table Experimental reflection coefficient (*C*_*R*_) for different *d/h* ratios. (DOCX)

S3 Table Experimental energy loss coefficient (*C*_*L*_) for different *d/h* ratios. (DOCX)

S4 Table Empirical transmission coefficient (*C*_*T*_) for different *d/h* ratios, calculated from the empirical model. (DOCX)

S5 Table Empirical reflection coefficient (*C*_*R*_) for different *d/h* ratios, calculated from the empirical model. (DOCX)

S6 Table Empirical energy loss coefficient (*C*_*L*_) for different *d/h* ratios, calculated from the empirical model. (DOCX)

S7 Table Experimental and empirical transmission coefficient (*C*_*T*_) values across different *d/h* ratios. (DOCX)

S8 Table Experimental and empirical reflection coefficient (*C*_*R*_) values across different *d/h* ratios. (DOCX)

S9 Table Experimental and empirical energy loss coefficient (*C*_*L*_) values across different *d/h* ratios. (DOCX)

## Abbreviations

B: Breakwater width
C_L_: Energy dissipation coefficient
C_R_: Wave reflection coefficient
C_T_: Wave transmission coefficient
d: Water depth
h: Height of the breakwater
H_i_: Average height of incident waves
H_R_: Average height of reflected waves
H_T_: Average height of waves transmitted
L: Length of a wave in units of its period, *T*
T: Period of waves

## References

[1] Valiela I., Bowen J.L., and York J.K., J.B. Mangrove Forests: One of the World’s Threatened Major Tropical Environments: At least 35% of the area of mangrove forests has been lost in the past two decades, losses that exceed those for tropical rain forests and coral reefs, two other well-known threatened environments. 2001. 51(10): p. 807–815.

[2] Duke N.C., et al., *A world without mangroves*? 2007. 317(5834): p. 41–42. 10.1126/science.317.5834.41b 17615322

[3] Jin-Eong O., J.H. The ecology of mangrove conservation & management. 1995. 295: p. 343–351.

[4] Kamali B. and Hashim R., J.E.E. Mangrove restoration without planting. 2011. 37(2): p. 387–391.

[5] Fitri A., Yao L., and Sofawi B. Evaluation of mangrove rehabilitation project at Carey Island coast, Peninsular Malaysia based on long-term geochemical changes. in *IOP Conference Series*: *Earth and Environmental Science*. 2019. IOP Publishing.

[6] Cheng L.S., *Application of Geotube Breakwater for Muddy Coastline Protection in Peninsular Malaysia*. 2018, University of Malaya (Malaysia).

[7] Sulaiman, R.B.R. and F.S.M. Mohidin. *Establishment of shoreline buffer zone through rehabilitation of degraded coastal mangroves*. in *MATEC Web of Conferences*. 2018. EDP Sciences.

[8] Saadon M.S. *Wave Transmission and Reflection of the Alternatively Submerged Geotube Breakwater*. 2016. IRC.

[9] Lee S.C., et al., *Utilization of geotextile tube for sandy and muddy coastal management*: *A review*. 2014. 2014. 10.1155/2014/494020 PMC4052796 24955408

[10] Takahashi S., *Design of vertical breakwaters*. 2002, Citeseer.

[11] Yuan D. and Tao J., J.C.E. Wave forces on submerged, alternately submerged, and emerged semicircular breakwaters. 2003. 48(2): p. 75–93.

[12] Graw K., et al., Dynamic pressures exerted on semicircular breakwater. Leipzig Annual Civil Engineering Report, 1998. 3: p. 333–344.

[13] Tanimoto K. Japanese experiences on composite breakwaters. in *Proc*. *Inter*. *Workshop on Wave Barriers in Deepwaters (Yokosuka*, *Japan)*, *1994*. 1994.

[14] Zhang N.-C., Wang L.-Q., and Yu Y.-X., J.C.e.j. Oblique irregular waves load on semicircular breakwater. 2005. 47(04): p. 183–204.

[15] Liu Y. and Li H.-J., J.A.O.R. Analysis of wave interaction with submerged perforated semi-circular breakwaters through multipole method. 2012. 34: p. 164–172.

[16] Ursell F. Surface waves on deep water in the presence of a submerged circular cylinder. I. in *Mathematical proceedings of the Cambridge philosophical society*. 1950. Cambridge University Press.

[17] Thorne R. Multipole expansions in the theory of surface waves. in *Mathematical Proceedings of the Cambridge Philosophical Society*. 1953. Cambridge University Press.

[18] Linton C.M. and McIver P., *Handbook of mathematical techniques for wave/structure interactions*. 2001: Chapman and Hall/CRC.

[19] Liu Y. and Li H.J., J.J.o.E.M. Analysis of oblique wave interaction with a submerged perforated semicircular breakwater. 2013. 83: p. 23–36.

[20] Liu Y., Li H.-J., and J.G.A.F. Dynamics, Oblique flexural-gravity wave scattering by a submerged semi-circular ridge. 2016. 110(3): p. 259–273.

[21] Li A.-j., Liu Y., and Lyu Z.-r., J.P.o.t.I.o.M.E. Part M: Journal of Engineering for the Maritime Environment, Analysis of water wave interaction with a submerged quarter-circular breakwater using multipole method. 2020. 234(4): p. 846–860.

[22] Teh H.M., et al., *Interaction of Waves with a Free-Surface Semicircular Breakwater*: *Experimental Investigation and Empirical Models*. 2023. 11(7): p. 1419.

[23] Roslan, M.N.A.B., H.M. Teh, and F.A.H. Al-Towayti. *Numerical Simulations of Wave Diffraction Around a Low-Crested Semicircular Breakwater*. in *Proceedings of the 5th International Conference on Water Resources (ICWR)–Volume 1*: *Current Research in Water Resources*, *Coastal and Environment*. 2022. Springer.

[24] Burcharth, H. and S.A. Hughes, *Fundamentals of design*, in *Coastal engineering manual*. 2003, Coastal Engineering Research Center. p. VI-5-i-VI-5-316.

[25] Cheng C.-L. and Garg G., J.J.o.M.A. Coefficient of determination for multiple measurement error models. 2014. 126: p. 137–152.

[26] Foo K.Y. and Hameed B.H., J.C.e.j. Insights into the modeling of adsorption isotherm systems. 2010. 156(1): p. 2–10.

[27] Altowayti W.A.H., et al., *Adsorption of Zn2+ from synthetic wastewater using dried watermelon rind (D-WMR)*: *an overview of nonlinear and linear regression and error analysis*. 2021. 26(20): p. 6176. 10.3390/molecules26206176 PMC8539476 34684757

[28] Willmott C.J. and Matsuura K., J.C.r. Advantages of the mean absolute error (MAE) over the root mean square error (RMSE) in assessing average model performance. 2005. 30(1): p. 79–82.

[29] Chai T. and Draxler R.R., J.G.m.d. Root mean square error (RMSE) or mean absolute error (MAE)?–Arguments against avoiding RMSE in the literature. 2014. 7(3): p. 1247–1250.

[30] Schlurmann T., Bleck M., and Oumeraci H., *Wave transformation at artificial reefs described by the Hilbert-Huang transformation (HHT)*, *in Coastal Engineering 2002*: *Solving Coastal Conundrums*. 2003, World Scientific. p. 1791–1803.

[31] Nguyen H.P., et al., *Representative transmission coefficient for evaluating the wave attenuation performance of 3D floating breakwaters in regular and irregular waves*. 2021. 9(4): p. 388.

[32] Dhinakaran G., et al., *Regular wave measurements on a submerged semicircular breakwater*. 2010. 132(3).

[33] Mahmoudi A., et al., *Experimental Study on Wave Transmission and Reflection at Impermeable Submerged Breakwaters*. 2017. 2(3): p. 19–27.

[34] Teh H.M. and Venugopal V., J.O.e. Performance evaluation of a semicircular breakwater with truncated wave screens. 2013. 70: p. 160–176.

[35] Young D.M. and Testik F.Y., J.O.E. Wave reflection by submerged vertical and semicircular breakwaters. 2011. 38(10): p. 1269–1276.

[36] Hegde A.V., Rao S., and Kumar K., J.I.J.o.H.E. Run-up, run-down and reflection characteristics of semicircular breakwater for varying seaside perforations. 2012. 18(3): p. 145–151.

[37] Hegde A.V., Naseeb S., and J.T.I.J.o.O.C. Systems, Transmission Performance of Submerged Semicircular Breakwaters for Different Radii and Submergence Ratios. 2014. 5(3): p. 151–161.

[38] Romya A.A., et al., *Performance assessment of corrugated semi-circular breakwaters for coastal protection*. 2022. 61(5): p. 3587–3598.

[39] Koutandos E. Hydrodynamic analysis of a skirt breakwater. in *Proceedings of the Institution of Civil Engineers-Maritime Engineering*. 2007. Thomas Telford Ltd.

[40] Günaydın K. and Kabdaşlı M., J.O.e. Performance of solid and perforated U-type breakwaters under regular and irregular waves. 2004. 31(11–12): p. 1377–1405.

[41] Günaydın K. and Kabdaşlı M., J.O.E. Investigation of Π-type breakwaters performance under regular and irregular waves. 2007. 34(7): p. 1028–1043.

[42] Dhinakaran, G., et al. *Pressures on a seaside perforated semicircular breakwater*. in *International Conference in Ocean Engineering- 2001*. 2001.

