# Correction: Hydrodynamic performance assessment of emerged and sub-merged semicircular breakwaters under random waves: An experimental and empirical study

**DOI:** 10.1371/journal.pone.0325159

**Published:** 2025-05-20

**Authors:** Faris Ali Hamood Al-Towayti, Hee Min Teh, Zhe Ma, Idris Ahmed Jae, Agusril Syamsir

The Acknowledgments section is omitted from the published article [1].

The correct Acknowledgments section is as follows: The authors would like to express their sincere gratitude to Yayasan Universiti Teknologi PETRONAS (YUTP) and the Ministry of Higher Education Malaysia (MOHE) for their generous financial support through grant 015LC0-352 (Performance characterization of an oscillating water column integrated with an operating or decommissioned offshore oil platform) and grant FRGS/1/2018/TK10/UTP/02/8, respectively.
