# Supplementary material for: Hydrodynamic performance assessment of emerged and sub-merged semicircular breakwaters under random waves: An experimental and empirical study
Source: PLoS One. 2025 Feb 3;20(2):e0313955. doi: 10.1371/journal.pone.0313955 (PMC11790088; doi:10.1371/journal.pone.0313955)
Supplement: S6 Table — (DOCX) [file pone.0313955.s006.docx]

**Table S6 - Empirical Energy Loss Coefficient (*C_L_*) Calculated from the Empirical Model**

|  | ***d/h =* 0.667** | | ***d/h =* 1.000** | | ***d/h =* 1.333** | | ***d/h =* 1.667** | |
| --- | --- | --- | --- | --- | --- | --- | --- | --- |
|  | ***B/L*** | ***C_L_* (Empirical)** | ***B/L*** | ***C_L_* (Empirical)** | ***B/L*** | ***C_L_* (Empirical)** | ***B/L*** | ***C_L_* (Empirical)** |
| ***H_i_/L* ≤ 0.02** | 1.22 | 0.73 | 1.20 | 0.71 | 1.20 | 0.52 | 1.20 | 0.15 |
|  | 0.82 | 0.63 | 0.95 | 0.66 | 0.95 | 0.49 | 0.77 | 0.17 |
|  | 0.71 | 0.62 | 0.66 | 0.64 | 0.77 | 0.50 | 0.64 | 0.19 |
|  | 0.62 | 0.61 | 0.57 | 0.65 | 0.64 | 0.51 | 0.54 | 0.21 |
|  | 0.55 | 0.61 | 0.50 | 0.65 | 0.55 | 0.52 | 0.46 | 0.23 |
|  | 0.50 | 0.61 | 0.44 | 0.66 | 0.47 | 0.53 | 0.40 | 0.25 |
|  | 0.46 | 0.61 | 0.40 | 0.67 | 0.42 | 0.54 | 0.36 | 0.27 |
|  | 0.42 | 0.61 | 0.37 | 0.67 | 0.37 | 0.55 | 0.32 | 0.27 |
|  | 0.39 | 0.62 | 0.34 | 0.67 | 0.34 | 0.56 | 0.29 | 0.29 |
|  | 0.37 | 0.62 | 0.31 | 0.68 | 0.31 | 0.56 | 0.27 | 0.30 |
|  | 0.34 | 0.62 | 0.29 | 0.68 | 0.29 | 0.57 | 0.25 | 0.31 |
|  | 0.31 | 0.62 | 0.28 | 0.68 | 0.26 | 0.58 | 0.23 | 0.31 |
|  | 0.29 | 0.62 | 0.26 | 0.69 | 0.25 | 0.58 | 0.22 | 0.32 |
|  | 0.28 | 0.63 | 0.25 | 0.69 | 0.23 | 0.59 | 0.20 | 0.33 |
|  | 0.26 | 0.63 | 0.23 | 0.70 | 0.22 | 0.60 | 0.19 | 0.34 |
|  |  |  | 0.22 | 0.70 | 0.21 | 0.60 | 0.18 | 0.35 |
|  |  |  |  |  | 0.20 | 0.60 | 0.17 | 0.35 |
| **0.02 < *H_i_/L* ≤ 0.04** | 1.22 | 0.71 | 1.20 | 0.70 | 1.20 | 0.52 | 1.20 | 0.16 |
|  | 0.98 | 0.66 | 0.78 | 0.65 | 0.95 | 0.50 | 0.95 | 0.17 |
|  | 0.82 | 0.64 | 0.66 | 0.66 | 0.77 | 0.52 | 0.54 | 0.26 |
|  | 0.71 | 0.63 | 0.57 | 0.67 | 0.64 | 0.54 | 0.46 | 0.29 |
|  | 0.62 | 0.63 | 0.50 | 0.68 | 0.55 | 0.55 | 0.40 | 0.30 |
|  | 0.55 | 0.63 | 0.44 | 0.69 | 0.47 | 0.57 | 0.36 | 0.32 |
|  | 0.50 | 0.63 | 0.40 | 0.69 | 0.42 | 0.58 | 0.32 | 0.33 |
|  | 0.46 | 0.63 | 0.37 | 0.70 | 0.37 | 0.59 | 0.29 | 0.34 |
|  | 0.42 | 0.64 | 0.34 | 0.70 | 0.34 | 0.60 | 0.25 | 0.35 |
|  | 0.39 | 0.65 | 0.31 | 0.70 | 0.31 | 0.60 | 0.23 | 0.37 |
|  | 0.37 | 0.66 | 0.29 | 0.71 | 0.29 | 0.61 | 0.22 | 0.38 |
|  | 0.34 | 0.66 | 0.28 | 0.72 | 0.26 | 0.61 | 0.20 | 0.40 |
|  | 0.32 | 0.66 | 0.26 | 0.72 | 0.25 | 0.63 | 0.19 | 0.41 |
|  | 0.31 | 0.66 | 0.25 | 0.73 | 0.23 | 0.63 | 0.18 | 0.43 |
|  | 0.29 | 0.67 | 0.23 | 0.74 | 0.22 | 0.64 |  |  |
|  | 0.28 | 0.68 |  |  | 0.21 | 0.65 |  |  |
|  |  |  |  |  | 0.20 | 0.66 |  |  |
| **0.04 < *H_i_/L* ≤ 0.06** | 1.22 | 0.71 | 1.20 | 0.70 | 1.20 | 0.53 | 1.20 | 0.18 |
|  | 0.98 | 0.67 | 0.95 | 0.67 | 0.77 | 0.53 | 0.95 | 0.20 |
|  | 0.82 | 0.65 | 0.78 | 0.67 | 0.64 | 0.55 | 0.77 | 0.22 |
|  | 0.55 | 0.66 | 0.66 | 0.70 | 0.47 | 0.59 | 0.64 | 0.25 |
|  | 0.50 | 0.67 | 0.57 | 0.70 | 0.42 | 0.63 | 0.54 | 0.28 |
|  | 0.46 | 0.67 | 0.50 | 0.71 | 0.37 | 0.63 | 0.46 | 0.32 |
|  |  |  | 0.44 | 0.73 | 0.34 | 0.64 | 0.40 | 0.36 |
|  |  |  | 0.40 | 0.74 | 0.31 | 0.64 | 0.36 | 0.37 |
|  |  |  | 0.37 | 0.74 | 0.29 | 0.67 | 0.32 | 0.38 |
|  |  |  | 0.34 | 0.75 | 0.26 | 0.68 | 0.29 | 0.40 |
|  |  |  | 0.31 | 0.75 | 0.25 | 0.69 | 0.27 | 0.42 |
|  |  |  | 0.29 | 0.75 | 0.23 | 0.70 | 0.25 | 0.44 |
|  |  |  | 0.28 | 0.77 | 1.20 | 0.53 | 0.22 | 0.45 |
|  |  |  | 0.26 | 0.78 | 0.77 | 0.53 |  |  |
