# Supplementary material for: Hydrodynamic performance assessment of emerged and sub-merged semicircular breakwaters under random waves: An experimental and empirical study
Source: PLoS One. 2025 Feb 3;20(2):e0313955. doi: 10.1371/journal.pone.0313955 (PMC11790088; doi:10.1371/journal.pone.0313955)
Supplement: S7 Table — (DOCX) [file pone.0313955.s007.docx]

**Table S7 - Experimental and Empirical Transmission Coefficient (*C_T_*) Values Across Different *d/h* Ratios**

|  | ***d/h =* 0.667** | | | ***d/h =* 1.000** | | | ***d/h =* 1.333** | | | ***d/h =* 1.667** | | |
| --- | --- | --- | --- | --- | --- | --- | --- | --- | --- | --- | --- | --- |
|  | ***B/L*** | ***C_T_* (Experimental)** | ***C_T_* (Empirical)** | ***B/L*** | ***C_T_* (Experimental)** | ***C_T_* (Empirical)** | ***B/L*** | ***C_T_* (Experimental)** | ***C_T_* (Empirical)** | ***B/L*** | ***C_T_* (Experimental)** | ***C_T_* (Empirical)** |
| ***H_i_/L* ≤ 0.02** | 1.22 | 0.04 | 0.14 | 1.20 | 0.33 | 0.45 | 1.20 | 0.92 | 0.84 | 0.77 | 0.99 | 0.99 |
|  | 0.82 | 0.02 | 0.02 | 0.95 | 0.38 | 0.49 | 0.95 | 0.91 | 0.85 | 0.64 | 0.99 | 0.97 |
|  | 0.71 | 0.03 | 0.01 | 0.66 | 0.40 | 0.54 | 0.77 | 0.90 | 0.85 | 0.54 | 0.98 | 0.96 |
|  | 0.62 | 0.03 | 0.03 | 0.57 | 0.46 | 0.55 | 0.64 | 0.86 | 0.85 | 0.46 | 0.98 | 0.94 |
|  | 0.55 | 0.03 | 0.04 | 0.50 | 0.51 | 0.55 | 0.55 | 0.84 | 0.85 | 0.40 | 0.97 | 0.93 |
|  | 0.50 | 0.04 | 0.05 | 0.44 | 0.57 | 0.56 | 0.47 | 0.84 | 0.85 | 0.36 | 0.94 | 0.92 |
|  | 0.46 | 0.04 | 0.06 | 0.40 | 0.58 | 0.56 | 0.42 | 0.87 | 0.85 | 0.32 | 0.96 | 0.92 |
|  | 0.42 | 0.07 | 0.07 | 0.37 | 0.63 | 0.57 | 0.37 | 0.87 | 0.85 | 0.29 | 0.93 | 0.92 |
|  | 0.39 | 0.07 | 0.08 | 0.34 | 0.64 | 0.57 | 0.34 | 0.90 | 0.85 | 0.27 | 0.92 | 0.91 |
|  | 0.37 | 0.08 | 0.08 | 0.31 | 0.63 | 0.57 | 0.31 | 0.86 | 0.85 | 0.25 | 0.89 | 0.91 |
|  | 0.34 | 0.07 | 0.09 | 0.29 | 0.62 | 0.57 | 0.29 | 0.87 | 0.85 | 0.23 | 0.87 | 0.90 |
|  | 0.31 | 0.08 | 0.09 | 0.28 | 0.63 | 0.57 | 0.26 | 0.86 | 0.84 | 0.22 | 0.84 | 0.90 |
|  | 0.29 | 0.08 | 0.09 | 0.26 | 0.61 | 0.57 | 0.25 | 0.83 | 0.84 | 0.20 | 0.81 | 0.89 |
|  | 0.28 | 0.06 | 0.10 | 0.25 | 0.59 | 0.57 | 0.23 | 0.81 | 0.84 | 0.19 | 0.84 | 0.88 |
|  | 0.26 | 0.04 | 0.10 | 0.23 | 0.57 | 0.57 | 0.22 | 0.78 | 0.84 | 0.18 | 0.83 | 0.88 |
|  |  |  |  | 0.22 | 0.55 | 0.57 | 0.21 | 0.80 | 0.84 | 0.17 | 0.87 | 0.87 |
|  |  |  |  |  |  |  | 0.20 | 0.85 | 0.84 |  |  |  |
| **0.02 < *H_i_/L* ≤ 0.04** | 1.22 | 0.02 | 0.10 | 1.20 | 0.29 | 0.47 | 1.20 | 0.92 | 0.84 | 0.95 | 0.95 | 0.99 |
|  | 0.98 | 0.01 | 0.03 | 0.78 | 0.35 | 0.52 | 0.95 | 0.92 | 0.85 | 0.54 | 0.98 | 0.92 |
|  | 0.82 | 0.01 | 0.01 | 0.66 | 0.42 | 0.54 | 0.77 | 0.87 | 0.85 | 0.46 | 0.97 | 0.90 |
|  | 0.71 | 0.02 | 0.04 | 0.57 | 0.45 | 0.55 | 0.64 | 0.86 | 0.84 | 0.40 | 0.96 | 0.89 |
|  | 0.62 | 0.02 | 0.05 | 0.50 | 0.51 | 0.55 | 0.55 | 0.83 | 0.84 | 0.36 | 0.95 | 0.89 |
|  | 0.55 | 0.03 | 0.06 | 0.44 | 0.58 | 0.55 | 0.47 | 0.81 | 0.83 | 0.32 | 0.93 | 0.88 |
|  | 0.50 | 0.03 | 0.07 | 0.40 | 0.60 | 0.55 | 0.42 | 0.82 | 0.83 | 0.29 | 0.91 | 0.87 |
|  | 0.46 | 0.03 | 0.08 | 0.37 | 0.65 | 0.56 | 0.37 | 0.82 | 0.82 | 0.25 | 0.88 | 0.87 |
|  | 0.42 | 0.06 | 0.09 | 0.34 | 0.62 | 0.56 | 0.34 | 0.81 | 0.82 | 0.23 | 0.86 | 0.86 |
|  | 0.39 | 0.06 | 0.09 | 0.31 | 0.60 | 0.56 | 0.31 | 0.79 | 0.82 | 0.22 | 0.82 | 0.85 |
|  | 0.37 | 0.09 | 0.10 | 0.29 | 0.59 | 0.56 | 0.29 | 0.76 | 0.82 | 0.20 | 0.80 | 0.84 |
|  | 0.34 | 0.09 | 0.10 | 0.28 | 0.60 | 0.56 | 0.26 | 0.76 | 0.82 | 0.19 | 0.88 | 0.84 |
|  | 0.32 | 0.08 | 0.10 | 0.26 | 0.58 | 0.56 | 0.25 | 0.73 | 0.81 | 0.18 | 0.86 | 0.83 |
|  | 0.31 | 0.08 | 0.11 | 0.25 | 0.67 | 0.56 | 0.23 | 0.72 | 0.81 |  |  |  |
|  | 0.29 | 0.09 | 0.11 | 0.23 | 0.67 | 0.56 | 0.22 | 0.72 | 0.81 |  |  |  |
|  | 0.28 | 0.10 | 0.11 | 1.20 | 0.29 | 0.49 | 0.21 | 0.73 | 0.81 |  |  |  |
|  |  |  |  |  |  |  | 0.20 | 0.78 | 0.80 |  |  |  |
| **0.04 < *H_i_/L* ≤ 0.06** | 1.22 | 0.01 | 0.05 | 0.95 | 0.90 | 0.52 | 1.20 | 0.91 | 0.86 | 0.95 | 0.96 | 0.99 |
|  | 0.98 | 0.00 | 0.00 | 0.78 | 0.37 | 0.54 | 0.77 | 0.88 | 0.85 | 0.77 | 0.99 | 0.96 |
|  | 0.82 | 0.01 | 0.04 | 0.66 | 0.44 | 0.56 | 0.64 | 0.83 | 0.84 | 0.64 | 0.97 | 0.93 |
|  | 0.55 | 0.03 | 0.09 | 0.57 | 0.48 | 0.56 | 0.47 | 0.75 | 0.83 | 0.54 | 0.98 | 0.91 |
|  | 0.50 | 0.03 | 0.10 | 0.50 | 0.53 | 0.56 | 0.42 | 0.74 | 0.82 | 0.46 | 0.95 | 0.89 |
|  | 0.46 | 0.04 | 0.11 | 0.44 | 0.56 | 0.56 | 0.37 | 0.75 | 0.82 | 0.40 | 0.94 | 0.88 |
|  |  |  |  | 0.40 | 0.60 | 0.56 | 0.34 | 0.72 | 0.81 | 0.36 | 0.94 | 0.87 |
|  |  |  |  | 0.37 | 0.77 | 0.56 | 0.31 | 0.71 | 0.81 | 0.32 | 0.87 | 0.86 |
|  |  |  |  | 0.34 | 0.77 | 0.56 | 0.29 | 0.79 | 0.81 | 0.29 | 0.84 | 0.85 |
|  |  |  |  | 0.31 | 0.73 | 0.56 | 0.26 | 0.77 | 0.80 | 0.27 | 0.80 | 0.84 |
|  |  |  |  | 0.29 | 0.76 | 0.56 | 0.25 | 0.73 | 0.80 | 0.25 | 0.77 | 0.84 |
|  |  |  |  | 0.28 | 0.70 | 0.56 | 0.23 | 0.69 | 0.80 | 0.22 | 0.75 | 0.83 |
|  |  |  |  | 0.26 | 0.64 | 0.56 |  |  |  |  |  |  |
